# Safety Evaluation of Lab-Made Clinoptilolite: 90-Day Repeated Dose Toxicity Study in Sprague Dawley Rats and a Battery of In Vitro and In Vivo Genotoxicity Tests

**DOI:** 10.3390/toxics14020122

**Published:** 2026-01-28

**Authors:** Polina Smith, Samit Kadam, Channaveerayya Mathada, Lauren Y. Park, Dylan Fronda, Moustafa Kardjadj

**Affiliations:** 1 Coseva Advanced Laboratories, Sandy, UT 84070, USA; 2 Vipragen Biosciences Private Limited, #67B, Hootagalli Industrial Area, Mysore 570018, Karnataka, India; 3 Dicentra, Toronto, ON M4W 3E2, Canada

**Keywords:** clinoptilolite, zeolite, genotoxicity, repeated-dose toxicity, safety assessment

## Abstract

Clinoptilolite is a zeolite with a microporous structure that enables ion exchange, molecular sieving, and adsorption, conferring detoxifying, antioxidant, and anti-inflammatory properties. These properties have applications in food, medicine, catalysis, and environmental remediation. This study evaluated the safety of the lab-made Clinoptilolite as a potential food ingredient through a 90-day repeated-dose toxicity study in male and female Sprague Dawley rats. The test substance was administered via oral gavage at doses of 0, 5, 10, and 15 mg/kg bw/day, followed by a 28-day recovery period. In addition, genotoxicity was assessed using the Ames test, in vitro chromosomal aberration assay, and an in vivo micronucleus test. All studies were conducted in accordance with OECD and FDA guidelines. Results showed no adverse systemic, genotoxic, or irreversible effects at any dose, with minor clinical variations being incidental and reversible. Genotoxicity tests confirmed no mutagenic or clastogenic potential. Overall, the lab-made Clinoptilolite evaluated in this investigation was well tolerated, non-toxic, and showed no evidence of treatment-related toxicity at the doses tested. These findings provide supportive evidence for its consideration toward a Generally Recognized as Safe (GRAS) determination.

## 1. Introduction

Clinoptilolite is a naturally occurring mineral belonging to the heulandite subgroup of zeolites, distinguished by its well-defined crystalline structure and microporous network of silica and alumina tetrahedra [1]. Like other aluminosilicate minerals, Clinoptilolite exhibits a unique structural framework that provides high surface area, ion-exchange capacity, and molecular sieving properties, making it useful in diverse applications such as catalysis, environmental remediation, medicinal, and food technology [2,3,4]. Its physicochemical properties enable the adsorption and exchange of water, ions, and other polar molecules, contributing to its potent detoxifying, antioxidant, and anti-inflammatory effects [5]. Several studies have demonstrated the potential beneficial effects of Clinoptilolite in vivo on mammals, highlighting its relevance as a natural therapeutic and functional material [6,7,8].

Mined Clinoptilolite typically exhibits lower purity because it forms alongside other minerals. In some deposits, the Clinoptilolite phase can be as low as 52.7%, with sanidine, cristobalite, mordenite, and muscovite occurring as accessory phases Supplementary Materials (Figures S1–S6). The lab-made Clinoptilolite crystal structure is identical to the Clinoptilolite found in nature. However, in contrast to the mined Clinoptilolite, the lab-made Clinoptilolite is produced under controlled conditions. It therefore shows much higher purity, reaching up to 95.2% in powder form and up to 99% when converted into a suspension, as confirmed by Coseva Advanced Laboratories unpublished XRD data that were conducted by a third-party lab Supplementary Materials (Figures S1–S6). In addition to achieving a higher clinoptilolite phase, another key advantage of lab-made over-minded Clinoptilolite is its significantly lower level of heavy-metal contaminants. Natural clinoptilolite’s strong ion-exchange properties and affinity for heavy metals result in mined material containing significant soil-derived impurities, necessitating rigorous decontamination before use [9,10]. The lab-made form avoids these issues, offering a cleaner, more controlled composition that is better aligned with the purity requirements expected for food-grade ingredients. Although many safety studies are available for mined Clinoptilolite, this study focuses specifically on the lab-made form found in the aqueous suspension, which differs in composition and size and thus warrants its own evaluation. As a candidate for functional food ingredient with physicochemical stability and biologically inert properties, it merits a detailed safety assessment to support Generally Recognized as Safe (GRAS) substantiation.

As part of pre-market safety assessment, new food ingredients undergo a thorough evaluation of their toxicological profile to confirm they present no potential for systemic, genetic, or other adverse effects when used as intended. Repeated-dose oral toxicity studies are a fundamental component of such evaluations, providing data on potential toxic effects by indicating major target organs and the identification of a no-observed-adverse-effect level (NOAEL). In addition, genotoxicity testing is critical to rule out the possibility of mutagenic or clastogenic effects, in accordance with international regulatory guidance [11,12]. To our knowledge, while toxicological studies have been reported for naturally occurring clinoptilolite, no published studies have evaluated the dietary use or conducted a formal toxicological assessment of lab-made Clinoptilolite.

The objective of this study was to assess the safety of the lab-made Clinoptilolite, prepared for this study, in its suspension form through a combination of toxicological approaches: A repeated-dose 90-day study in *Sprague Dawley* rats to assess systemic effects and potential toxicity and reversibility during 28-day post-treatment, along with a series of established genotoxicity tests, including the bacterial reverse mutation (Ames) test, the in vitro chromosomal aberration test in Chinese Hamster Ovary K-1 (CHO-K1) cells, and the in vivo micronucleus assay in *Swiss albino* mice. These studies were conducted in compliance with relevant OECD and FDA guidelines to support the GRAS safety substantiation of lab-made Clinoptilolite suspension as a food ingredient.

## 2. Materials and Methods

### 2.1. Test Item

The test item evaluated in this study was lab-made Clinoptilolite in its suspension form, supplied by Coseva Advanced Laboratories (Sandy, UT, USA; Lot no. RD102324). The test item was prepared using a methodology based on existing literature. The exact preparation details are maintained as a trade secret, though the process was conducted under controlled laboratory conditions to ensure batch consistency, quality, and reproducibility. The tested batch was intentionally selected to contain a lower proportion of clinoptilolite and higher levels of additional zeolitic phases to provide a conservative assessment of potential toxicity. The crystalline mineral phases were identified by x-ray diffraction (XRD) analysis of the dried suspension residue, confirming a phase composition of 90.2% Clinoptilolite, 5.6% mordenite, and 4.2% philipsite, and was supplied as a non-soluble suspension, as confirmed by a certificate of analysis (COA). Particle size distribution in the hydrated suspension was evaluated using dynamic light scattering (DLS), with an average hydrodynamic diameter of 229 nm determined by intensity-weighted analysis. Measurements were conducted on the aqueous suspension using Milli-Q water as the dispersant. Full DLS results are provided in the Supplementary Materials (Figures S1–S6). Zeta potential and suspension stability were not evaluated, as the test material was administered as a freshly prepared suspension by oral gavage and was not intended for characterization as a nanomaterial. For administration, prior to use, the suspension was shaken well to ensure homogeneity and minimize sedimentation. Formulations were stored at room temperature.

Milli-Q water was selected as the vehicle control and was administered without the active test substance to serve as a control for comparison. The pH of the vehicle and test material solutions was measured and confirmed. The test material was prepared at the intended dose levels; verification of the prepared doses was performed to ensure accuracy and consistency. The dose level was chosen as highest amount of test material that could be administered to the rats, balancing practical administration with animal welfare considerations.

### 2.2. Repeated-Dose Toxicity Study (OECD 408)

#### 2.2.1. Study Animals and Group Assignment

Healthy *Sprague Dawley* rats were used in this study. Animals were housed under controlled environmental conditions, temperatures ranging from 20.1 °C to 23.8 °C, relative humidity of 45 to 68%, and with artificial lighting of 12 h light and dark cycle, and animals were provided with a standard diet and water *ad libitum*. At treatment initiation, the animals were 6–7 weeks of age. The initial body weight ranges were 220.31–276.21 g for males and 159.06–198.92 g for females. The males were acclimatized for 6 days, and the females were acclimatized for 7 days to the laboratory conditions. Health and welfare were monitored daily, and veterinary care was provided in accordance with GLP standards.

At the end of the acclimatization period, healthy rats were weighed and grouped before the initiation of treatment and allocated to their respective treatment groups using a body weight-based stratified randomization method with validated MS Excel spreadsheets. For the main groups, a total of 80 animals were randomized into 4 different groups, with 20 animals per group (10 females and 10 males). This randomization ensured that baseline body weights did not differ statistically among the groups. The mean body weights of each group before the start of treatment did not exceed ±20% of the mean body weight in each sex and group. Dose levels of low-dose (5 mg/kg/day), mid-dose (10 mg/kg/day), and high-dose (15 mg/kg/day) were selected based on prior toxicity data and range-finding studies. Each group received one dose at approximately the same time each day via oral gavage. In the same manner, animals in the control group received milli-Q water for 90 consecutive days.

The recovery cohort consisted of 20 animals, with 10 animals (5 males and 5 females) assigned to the control and high dose (15 mg/kg/day) groups. Animals were observed for 28 days following the cessation of treatment, during which no doses were administered.

This study was performed at Vipragen Biosciences Private Limited (Karnataka, India), a CCSEA approved laboratory under Registration number 1683/PO/RcBiBt/S/13/CPCSEA following all ethical practices as laid down in the guideline/s for animal care and accredited by AAALAC International, USA. The study protocol was reviewed and approved by the test facility’s Institutional Animal Ethics Committee (IAEC) on 19 March 2024. Protocol approval number: VIP-IAEC-448-2024 and amendment to IAEC protocol no. VIP-IAEC-448-2024-PA01 and VIP-IAEC-448-2024-PA02.

#### 2.2.2. Design and Endpoints

Animals were observed twice daily for mortality and morbidity and once daily for general clinical signs, and detailed clinical examinations were performed before the initiation of treatment and weekly once thereafter. During detailed clinical examination, animals were observed for signs of toxicity, including changes in skin, fur, eyes, mucous membranes, secretions, gait, posture, behavior, and these observations were recorded throughout the treatment and recovery periods. Functional observation battery tests were conducted at the end of the treatment period and during recovery period in control and high dose group animals. Body weights were measured at baseline, pre-dose (day 1), and weekly once thereafter. Feed consumption was recorded weekly, and both individual and group intake per rat were calculated. At the end of the treatment period, Blood samples were collected through retro orbital sinus puncture method for the estimation of Hematology, coagulation, clinical chemistry, parameters and were analyzed using calibrated automated instruments. All values are reported as mean value and standard deviation (mean ± SD). Urine samples were collected from all main groups during Week 13 using individual metabolic cages following overnight fasting and analyzed using validated automated analyzers and dipsticks.

Following the 90-day dosing period, animals (10 rats from control and 10 rats from high -dose group) underwent a 28-day recovery period without treatment and were then sampled and necropsied as per the main study. Recovery groups were analyzed separately to evaluate the delayed occurrence of toxic effects or reversibility of any treatment-related effects. While in-life observations were conducted in an open-label manner according to OECD 408 guidelines, all subsequent histopathological evaluations were performed in a blinded manner to ensure objective assessment and eliminate potential observer bias.

### 2.3. Necropsy and Histopathological Evaluation

At study termination, animals were euthanized by exsanguination under CO_2_ asphyxiation. Gross pathology examinations were performed, and selected organs (liver, kidneys, spleen, heart, adrenals, thymus, brain, testes, ovaries, uterus with cervix, thyroid with parathyroid, and pituitary gland) were collected, weighed, and preserved. Relative organ weights calculated as (organ weight/terminal weight) × 100. Tissues were fixed in 10% neutral buffered formalin, paraffin-embedded, H&E-stained, and examined by a pathologist. Lesion incidence and severity were recorded.

### 2.4. Genotoxicity Study

#### 2.4.1. Ames Assay (OECD 471)

The bacterial reverse-mutation test (Ames test) was conducted using *Salmonella typhimurium strains* (TA98, TA100, TA1535, TA1537) and *E. coli* WP2 (pKM101) to cover frameshift and base-pair substitution mutations. Milli-Q water served as the vehicle control for all tester strains, with concurrent vehicle controls included for each strain. Positive controls consisted of sodium azide, 2-nitrofluorene, or ICR191 (without S9 activation, depending on the strain), and 2-aminoanthracene (for all strains with S9 activation).

Based on the preliminary cytotoxicity test, the following concentrations were selected for the main study: 5000, 1500, 500, 150, and 50 µg/plate for all strains, with and without S9 metabolic activation. For cytotoxic test items, testing was conducted up to the concentration at which cytotoxicity was observed, characterized by a reduction in spontaneous revertants or a diminished bacterial background lawn.

Metabolic activation was achieved using 10% (*v*/*v*) rat liver S9 fractions induced by sodium phenobarbital and β-naphthoflavone, prepared in-house (Vipragen Biosciences Pvt. Ltd. Karnataka, India). The 10 mL S9 mixture contained Milli-Q water (3.35 mL), 100 mM sodium phosphate buffer (pH 7.4) (5.00 mL), 4 mM NADP (0.40 mL), 8 mM MgCl_2_ (0.20 mL), 5 mM D-glucose-6-phosphate (0.05 mL), and rat S9 fraction (1.0 mL).

Plates were prepared in triplicate and incubated using standard plate incorporation and pre-incubation method per OECD 471 test guidelines. A test item was considered mutagenic if there was a concentration-related increase in revertants or a reproducible increase in at least one strain, with or without S9. Specifically, a two-fold increase for TA98, TA100, and E. coli WP2, and a three-fold increase for TA1535 and TA1537 compared to vehicle controls, was required for a positive response. Results are presented as mean revertant counts ± SD for each vehicle control, treatment group and positive control.

#### 2.4.2. In Vivo Micronucleus Test (OECD 474)

A dose range-finding study (DRF) was conducted to identify study-limiting toxicity and select appropriate doses for the main study, with a dose range of 250–2000 mg/kg bw/day. *Swiss albino* mice were administered 2000 mg/kg of Clinoptilolite by oral gavage once daily for 2 consecutive days. The study included 3 groups: a vehicle control group, a dose group, and a positive control group. Cyclophosphamide (50 mg/kg/day) was used as the positive control. The combined sample size for both the DRF and main study was 60 mice in total, with 3 males and 3 females in each of the 5 dose groups for the DRF phase, and 5 males and 5 females in each of the 3 test groups for the main study. Bone marrow samples were collected 24 h after the final dose, and the percentage of micronucleated polychromatic erythrocytes (%MnPCE) and the percentage of polychromatic erythrocytes (%PCE) were determined. The primary endpoint was the ratio of polychromatic erythrocytes to total erythrocytes (PCE/TE). The study was conducted in accordance with OECD 474 Test Guideline.

#### 2.4.3. In Vitro Mammalian Chromosomal Aberration (OECD 473)

CHO-K1 cells were employed for the study, conducted under Good Laboratory Practice (GLP) conditions in accordance with OECD Test Guideline 473. Cytotoxicity was evaluated at concentrations ranging from 31.25 to 2000 µg/mL using three regimens: a short-term treatment (3 h) with or without S9 metabolic activation, and a long-term treatment (20 h) without S9. Positive controls included Cyclophosphamide monohydrate (6 µg/mL) for metabolic activation and Mitomycin-C (0.3 µg/mL) for the absence of metabolic activation. The main study tested concentrations of 500, 1000, and 2000 µg/mL, both with and without S9 metabolic activation, and a total of 300 well-spread metaphase cells were scored for each treatment concentration. The primary endpoint was the frequency of aberrant cells (%), calculated based on the total number of metaphases scored.

### 2.5. Data Analysis

All statistical analyses were performed using R (R Foundation for Statistical Computing) with standard analytical packages. Mixed models and adjusted means were calculated using lme4, lmerTest, and emmeans, while Type III ANOVA was conducted with the car package. One-way ANOVA and post hoc comparisons were performed using base R functions. A 2-sided significance level of α = 0.05 was applied throughout. When multiple pairwise comparisons were made against the control group, Dunnett’s test (or an equivalent method) was used to control the familywise errors. Data distributions and variance assumptions were evaluated graphically and with Shapiro–Wilk and Levene’s tests. When assumptions were not met, data were log-transformed, rank-transformed, or analyzed using appropriate non-parametric tests. The specific statistical method applied to each parameter is indicated in the corresponding subsection of the Methods.

#### 2.5.1. Body Weight Time Course (Repeated Measures)

Body weight (BW) trajectories were analyzed separately by sex using a linear mixed-effects model (LMM) of the form lmer(bw_g ~ factor(dose) × day + (day|animal_id)). This specification included treatment group, time (day), and their interaction as fixed effects, with a random intercept and slope for each animal to account for repeated measures. The primary inferential test was the treatment × time interaction; if this interaction was not significant, the main effects of treatment and time were evaluated. Model-derived predicted body-weight trajectories and associated 95% confidence intervals (CI) were generated using emmeans for visualization.

#### 2.5.2. Feed Consumption

Feed consumption was analyzed using an analogous approach to body-weight data. When per-animal time-series measurements were available, feed intake was evaluated using linear mixed-effects models (LMMs), with animal included as a random effect to account for repeated measures. Fixed effects included treatment group and time, as well as their interaction. In cases where only aggregated data were available, mean weekly feed consumption was compared across treatment groups using a one-way ANOVA followed by Dunnett’s post hoc test to assess pairwise differences relative to the control group.

#### 2.5.3. Terminal Clinical Pathology (Hematology and Chemistry)

Terminal hematology and clinical chemistry parameters were compared across dose groups using a one-way ANOVA (factor = dose) with Dunnett’s post hoc tests versus the control group when parametric assumptions were satisfied. When parametric assumptions were not met, a Kruskal–Wallis test was applied, followed by appropriate non-parametric pairwise comparisons. For pre-specified analytes, relevant covariates (e.g., hematocrit or sex) were included in the model. Recovery groups were analyzed separately from the main study groups.

#### 2.5.4. Organ Weights

Since organ weight correlates with body weight, absolute organ weights were analyzed by ANOVA (organ weight as dependent variable; factor(dose) as fixed effect; terminal body weight as covariate). Adjusted means and 95% CI were computed using emmeans. Relative organ weights (organ ÷ terminal BW × 100) are reported for completeness and visualized.

#### 2.5.5. Ames Test

Revertant colony counts were analyzed separately for each tester strain. Both predefined fold-increase criteria and statistical significance tests were applied to assess potential treatment-related increases relative to the vehicle control. Positive controls met all assay acceptance criteria.

#### 2.5.6. Micronucleus Assay

For the micronucleus endpoint, mean ± SD values for MnPCE were summarized by the group. Treatment groups were compared with the vehicle control using either a *t*-test or a non-parametric alternative. A positive control group was included to verify assay sensitivity and expected responsiveness.

#### 2.5.7. Chromosomal Aberration Test

Structural chromosomal aberrations were enumerated for each group, with gaps excluded from the analysis. Group comparisons were conducted using Chi-square or Fisher’s exact tests, as appropriate, with a significance threshold of α = 0.05.

#### 2.5.8. Software and Plotting

Analyses were performed in R (version 4.5.1; R Foundation for Statistical Computing). Key packages included lme4, lmerTest, and emmeans for modeling and estimated marginal means; car for Type III ANOVA; and ggplot2 and ggpubr for data visualization. Figures were generated within R and exported as high-resolution PNG files (300 dpi) with white backgrounds to meet journal submission requirements.

## 3. Results

### 3.1. Repeated-Dose Toxicity Evaluation

**Figure 1:**
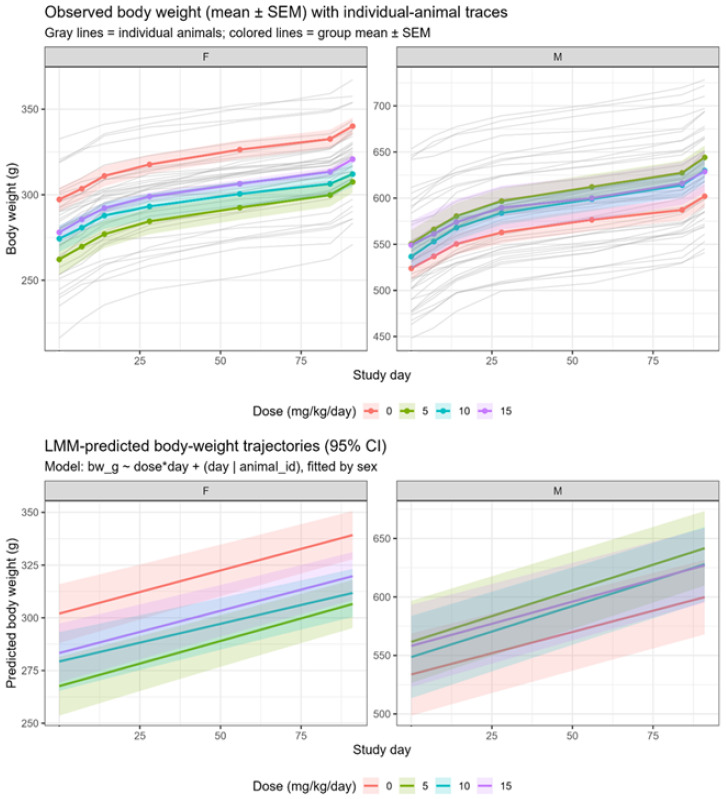
Body weight time course by sex and dose. (**Top**) Observed body weight (mean ± SEM) with individual animal traces shown as faint gray lines to illustrate within-group variability. (**Bottom**) Predicted body weight trajectories (95% CI) based on linear mixed-model (LMM) analysis. The LMM showed no consistent treatment-by-time interaction. BW: body weight; CI: confidence interval; LMM: linear mixed-model; SEM: standard error of the mean.

**Figure 2:**
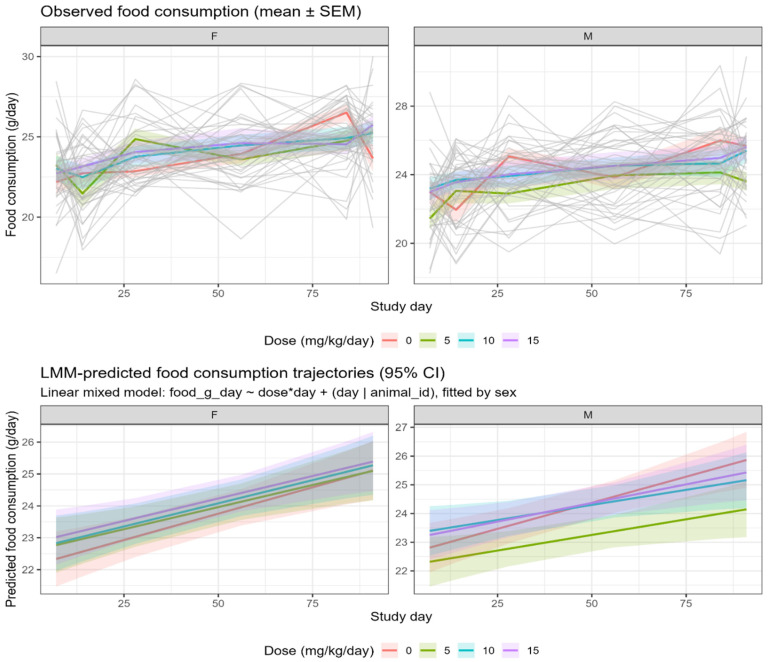
Food consumption time course by sex and dose in Sprague–Dawley rats ($n = 10$ per sex per group; 80 total). (**Top**) Observed food consumption (mean ± SEM) with individual animal traces shown as faint gray lines to illustrate within-group variability. (**Bottom**) Predicted food consumption trajectories (95% CI) based on linear mixed-model (LMM) analysis. The LMM showed no significant treatment-related changes. CI: confidence interval; LMM: linear mixed-model; SEM: standard error of the mean.

Repeated oral administration of Clinoptilolite at doses up to 15 mg/kg/day for 90 days in Sprague–Dawley rats did not show any treatment-related effects as there are no mortality and clinical signs of toxicity observed in any of the dose group or either sex throughout the treatment and recovery period. Clinical evaluations of all 80 animals (20 per group; 10 per sex) showed normal findings, with no abnormalities observed in any of the assessed parameters (Table 1). No biologically meaningful or dose-related effects on body weight were identified. LMM indicated no consistent treatment-by-time interaction suggestive of an adverse effect (Figure 1). Terminal fasting body weight comparisons showed a statistically significant differences in low-dose males relative to controls (*p* = 0.041). No other treatment groups showed significant differences (Table 2). Notably, sporadic statistical differences in net body weight gain occurred between Days 50 and 57, consisting of an increase in low-dose males and a decrease in mid-dose females. These fluctuations did not follow a dose–response pattern and occurred without corresponding changes in feed consumption. Overall feed-consumption assessments demonstrated no treatment changes (Figure 2), with weekly feed-consumption data remaining consistent across all groups throughout the study (Table 3 and Table 4).

### 3.2. Clinical and Histopathology Toxicity Evaluation

In the repeated-dose toxicity study, hematology parameters showed statistically significant changes in specific groups. In males, statistically significant decreases in red blood cell (RBC) count, hemoglobin (Hgb), and hematocrit (HCT), and an increase in mean corpuscular hemoglobin concentration (MCHC), were observed in the 5 mg/kg/day group compared with the vehicle control. In females, a statistically significant increase in eosinophil percentage was observed in the 5 mg/kg/day group. (Table 5 and Table 6). In clinical chemistry, males showed a statistically significant decrease in sodium in the 5 mg/kg/day group, an increase in glucose in the 10 mg/kg/day group, and increases in total protein, albumin, and calcium in the 15 mg/kg/day group. In females, statistically significant decreases in total bilirubin in the 5 mg/kg/day group and sodium in the 15 mg/kg/day group, as well as increases in urea and blood urea nitrogen (BUN) in the 15 mg/kg/day group, were recorded (Table 7).

Urinalysis parameters showed no treatment-related changes. Mean values for urine volume, specific gravity, and pH were comparable across all groups. Trace protein or epithelial cells were observed at low incidence across groups without a dose-related pattern, and all urinalysis values remained within normal physiological limits (Table 8). No dose-related trends were observed in these findings.

Organ weight evaluations did not show any findings that indicated adverse treatment-related effects. In males, absolute liver weight in the group that received 5 mg/kg/day was statistically higher than in controls. After adjustment using ANCOVA, no findings indicated adverse effects on liver weight. In females, both absolute and relative spleen weights in the high-dose group (15 mg/kg/day) were statistically significantly higher than those of the control group. No other consistent or meaningful changes in organ weights were observed across the remaining female groups. (Table 9 and Table 10). Microscopic examination of the liver, kidney, spleen, thyroid, and other organs revealed no treatment-related lesions. All microscopic findings were consistent with incidental or spontaneous background changes and did not show any relationship to dose (Table 11).

### 3.3. Genotoxicity

#### 3.3.1. Ames Bacterial Reverse-Mutation Assay

The test item was evaluated in the Ames bacterial reverse mutation assay using five tester strains (TA98, TA100, TA1535, TA1537, and *E. coli* WP2) with and without metabolic activation, under either metabolic activation condition (−S9 or +S9) in both Trial 1 (plate-incorporation) and Trial 2 (pre-incubation). At the highest concentration tested (5000 µg/plate), the test item did not produce any biologically or statistically relevant increase in revertant colonies in any strain under either metabolic condition. Mean revertant counts for the test item were comparable to concurrent vehicle controls and fell within laboratory historical control ranges (Table 12).

All positive controls (2-NF, 4-NQO, sodium azide, ICR-191, 2-AA) produced robust increases in revertants, confirming assay sensitivity and validity.

#### 3.3.2. In Vivo Micronucleus Assay (OECD 474)

The in vivo micronucleus assay was conducted to evaluate the clastogenic potential of Clinoptilolite by measuring its ability to induce micronuclei formation in immature erythrocytes following oral (gavage) administration to *Swiss albino* mice. Under the conditions of this study (oral dosing, bone marrow sampled 24 h after the last dose), the test item did not induce a biologically or statistically significant increase in micronucleated immature erythrocytes in *Swiss albino* mice at the maximum tested dose of 2000 mg/kg (Table 13). The positive control response and %PCE data indicate adequate assay performance and bone-marrow exposure.

#### 3.3.3. In Vitro Chromosomal Aberration Assay (OECD 473)

**Figure 3:**
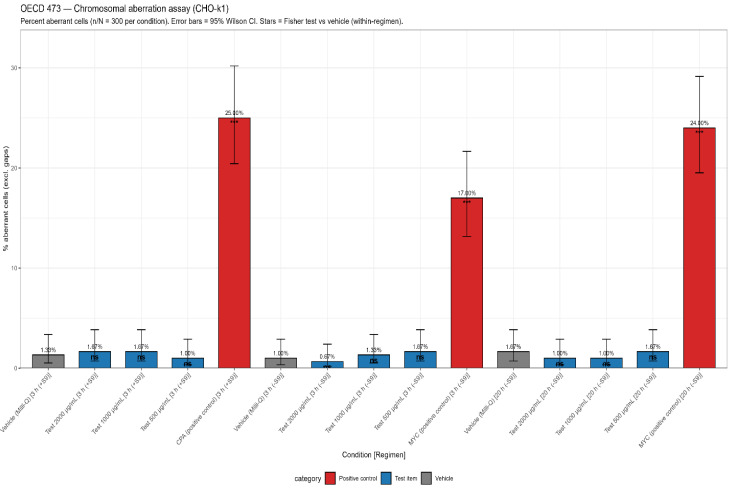
In vitro chromosomal aberration test results (OECD 473): Test system was Chinese hamster ovary cells (CHO-K1); 300 metaphases scored per condition. No test-item–related increase in cells with structural chromosomal aberrations was observed under any treatment conditions. *** statistically significant.

An in vitro mammalian chromosomal aberration test was conducted to evaluate the structural chromosomal aberrations in cultured mammalian cells (CHO-K1) exposed to the test item with and without metabolic activation (rat liver S9). Under the conditions of the OECD 473 test, the test item did not induce a biologically or statistically significant increase in cells with structural chromosomal aberrations in CHO-K1 cells up to 2000 µg/mL, either in the presence or absence of metabolic activation (Figure 3). The strong responses of the positive controls (CPA and mitomycin C) confirmed assay validity.

### 3.4. Recovery Phase Evaluation (Post-Treatment)

Twenty recovery animals were selected from each of the Control (n = 10; 5 males and 5 females) and High-Dose Clinoptilolite 15 mg/kg/day (n = 10; 5 males and 5 females) groups. These animals received 90 days of repeated dosing and were observed for an additional 28 days to assess recovery. Body weight and feed consumption in recovery group remained comparable to the control-recovery group, indicating no persistent effects (Table 14). Hematology and clinical chemistry values (means ± SD) in the recovery groups showed that changes observed at termination, such as slight increases in BUN or albumin, were no longer present; values were comparable between control-recovery and high-dose-recovery animals (Table 15). Post-treatment organ weights and histopathology assessments in recovery groups revealed no persistent microscopic lesions attributable to treatment, and organ weights were comparable to control-recovery animals (Table 16).

## 4. Discussion

### 4.1. Repeated-Dose Toxicity Evaluation

Repeated oral administration of the Clinoptilolite to Sprague Dawley rats for 90 days resulted in no mortality or treatment-related clinically significant findings in either sex, indicating that the compound was well tolerated at all tested doses. Mean body weights and feed consumption remained comparable to those of the vehicle control group throughout the study.

During the 90-day observation period, although a statistically significant increase in net body weight gain was noted in low-dose males (5 mg/kg/day; 28.97 g) and a decrease occurred in mid-dose females (10 mg/kg/day; 7.00 g) between Days 50 and 57, these were considered incidental. These fluctuations were transient, lacked a clear dose–response relationship, and occurred without corresponding changes in feed consumption or clinical or pathological correlates. Therefore, they were considered incidental and within the range of normal biological variation. All findings observed at the end of the dosing phase were reversible or absent following a 28-day recovery period, with no statistically significant differences between test item recovery groups and control. Throughout this study, some endpoints showed statistically significant differences compared to controls; however, in the absence of dose–response relationships, histopathological correlates, or values outside historical control ranges, such changes are considered incidental and not toxicologically meaningful. This distinction between statistical significance and biological relevance is applied throughout the evaluation of repeated-dose toxicity endpoints.

### 4.2. Clinical and Histopathological Toxicity Evaluation

Clinical pathology evaluations, including hematology and urinalysis, revealed no test item–related changes in toxicological significance in either sex. In the male group, a statistically significant decrease in RBC, Hgb, and HCT, and an increase in MCHC, were observed in the low-dose group (5 mg/kg/day) compared with the vehicle control. In females, a statistically significant increase in eosinophil percentage was also noted at 5 mg/kg/day dosed compared with controls. These findings were deemed incidental due to the absence of a consistent dose–response relationship and their occurrence only at isolated dose levels. In male animals, a statistically significant decrease in sodium at 5 mg/kg/day, an increase in glucose at 10 mg/kg/day, and increases in total protein, albumin, and calcium at 15 mg/kg/day were observed when compared with the vehicle control group. In females, statistically significant decreases in total bilirubin in low-dose group and sodium in high-dose group and increases in urea and BUN in high-dose group were recorded. The changes in total protein, albumin, and calcium in males and the changes in urea, BUN, and sodium in females were minimal, lacked dose dependency, and were not accompanied by corresponding alterations in organ weights, gross pathology, or histopathology. Therefore, these variations were deemed incidental and unrelated to Clinoptilolite administration. Overall, although a small number of parameters were statistically significant (*p* < 0.05) in isolated instances, none demonstrated a clear dose–response trend or associated morphological correlates. In recovery males, a statistically significant decrease in potassium and increases in urea and BUN were observed at 15 mg/kg/day compared with the controls. These variations were incidental, as they were minimal in magnitude, lacked dose dependency, and were not observed in the main study groups. In recovery females, a statistically significant increase in absolute spleen weight was noted in the high-dose group compared with controls. This change is also regarded as incidental due to the absence of corresponding gross or histopathological findings, and is therefore of no toxicological signification. Urinalysis parameters were likewise unremarkable, with no test item–related effects observed in either sex in the main or recovery groups, indicating no evidence of renal or metabolic disturbance attributable to treatment.

Comprehensive necropsy, organ weight assessment, and histopathological evaluation of major organs revealed no evidence of systemic or target organ toxicity. In males, a statistically significant increase in both absolute and relative liver weights was observed in the low-dose group compared with the vehicle control. In females, both absolute and relative spleen weights were significantly higher in the high-dose group (15 mg/kg/day). However, because no corresponding lesions were observed during histopathological examination, these increases were not considered an adverse effect of Clinoptilolite treatment. The changes in liver weight in males were deemed incidental in the absence of a dose-dependent trend or correlating microscopic alterations. Similarly, the increased spleen weight in high-dose females was regarded as incidental, given the lack of corresponding gross or histopathological changes. Therefore, these variations are deemed unrelated to Clinoptilolite administration. Microscopic findings observed across examined tissues were limited to incidental spontaneous lesions typically encountered in rats of this strain and age, without evidence of a dose-related pattern. No test item-related histopathological changes were identified in any organs or tissues examined. Lesions noted in the liver, kidneys, lungs, heart, adrenals, testes, and epididymides of both control and 15/mg/kg group were consistent with background findings and are toxicologically irrelevant. No treatment-related changes were observed during the qualitative assessment of spermatogenesis stages or in the morphological evaluation of interstitial testicular cell structures in high-dose males compared with controls. Similarly, the stage of the Estrous cycling in females from the control and high-dose groups at terminal sacrifice correlated appropriately with the histological appearance of the reproductive organs, indicating no adverse effects on reproductive tissues. Taken together, these results confirm the systemic tolerability of Clinoptilolite via oral administration, with no adverse effects observed up to the highest tested dose of 15 mg/kg/day. These findings demonstrate a wide margin of safety for the test item under the conditions of the present study. The complete resolution of findings during the recovery phase also highlights the physiological adaptability of the test animals and indicates that Clinoptilolite does not bioaccumulate or induce cumulative toxicity following repeated oral exposure.

### 4.3. Genotoxicity Evaluation

The results of the genotoxicity testing further support the benign safety profile of Clinoptilolite. The test item was negative in the standard battery of assays, including the Ames test, the in vitro chromosomal aberration test in CHO-K1 cells, and the in vivo micronucleus assay in mice. The results of the Ames test for Clinoptilolite clearly indicate that the test substance does not possess mutagenic potential under experimental conditions. Using *Salmonella typhimurium* strains TA98, TA100, TA1535, and TA1537, along with *E. coli* WP2 (pKM101), Clinoptilolite was tested at concentrations ranging from 50 to 5000 µg/plate, both with and without metabolic activation (S9 mix). The absence of cytotoxicity and the lack of any increase in revertant colony numbers across all strains and concentrations demonstrate that Clinoptilolite did not cause any mutations. Furthermore, the vehicle control results were consistent with historical spontaneous revertant ranges, confirming the validity of the assay, while the positive controls produced the expected responses, demonstrating appropriate test sensitivity and metabolic activation. Collectively, these findings confirm that Clinoptilolite is non-mutagenic in bacterial systems up to the highest concentration tested (5000 µg/plate).

The in vivo micronucleus test conducted with Clinoptilolites in *Swiss albino* mice demonstrated that the compound does not induce chromosomal damage in bone marrow cells. In the preliminary dose range-finding study, only minimal toxicity was observed, with slight percentage reductions across doses from 250 to 2000 mg/kg/day, indicating good tolerability. During the main study, mice administered Clinoptilolite orally at 2000 mg/kg/day for 2 consecutive days did not induce the formation of MnPCE in bone marrow, and the mean MnPCE value (2.5) was comparable to the negative control and substantially lower than that of the positive control (14.8). The small decrease in the PCE/TE ratio at the highest dose was not biologically relevant, confirming limited cytotoxicity in bone marrow cells. Additionally, no external or internal abnormalities were noted upon necropsy. The validity of the assay was confirmed by the expected response in the positive control group. Overall, these findings indicate that Clinoptilolite did not cause micronucleus formation or chromosomal damage in vivo, suggesting it is non-genotoxic at doses up to 2000 mg/kg body weight/day.

The in vitro chromosomal aberration test conducted with Clinoptilolite in cultured CHO-K1 mammalian cells demonstrated that the compound does not induce chromosomal abnormalities. Cytotoxicity assessments across concentrations from 500 to 2000 µg/mL showed minimal effects, with only 2.80–5.89% reduction in cell viability, while positive controls (Cyclophosphamide and Mitomycin-C) produced the expected 19–23% cytotoxicity, confirming assay validity. The short-term (3 h) and long-term (20 h) treatments test, both with and without S9 metabolic activation, showed comparable results between the Clinoptilolite-treated groups (0.67–1.67%) were comparable to vehicle controls (1.00–1.67%) in frequency of aberrant cells and showed no statistically significant or dose-dependent increase. Positive controls exhibited the expected high frequency of aberrations (17–25%), further validating the assay. Overall, these findings indicate that Clinoptilolite did not cause structural chromosomal damage in CHO-K1 cells at any tested concentration, either in the presence or absence of metabolic activation, confirming its non-clastogenic potential. These findings demonstrate that Clinoptilolite is non-mutagenic and non-clastogenic under the conditions tested. Collectively, the study outcome is consistent with the established understanding that clinoptilolite is chemically stable, maintains structural integrity under physiological conditions, and possesses a porous framework that allows ion exchange without altering its crystalline structure, supporting its suitability for in vivo applications [7,13]. The overall study design and conduct adhered to internationally accepted guidelines, including OECD Test Guidelines 408, 471, 473, and 474, and complied with Good Laboratory Practice (GLP) principles. These protocols align with the toxicological principles of the U.S. FDA Redbook for evaluating the safety of food and dietary ingredients. The absence of adverse systemic or genetic effects across all endpoints fulfills the toxicological expectations for substances considered for GRAS status or equivalent regulatory categories.

### 4.4. Perspectives and Limitations

The highest dose tested (15 mg/kg/day) produced no adverse effects and was therefore identified as the NOAEL. The NOAEL provides a substantial margin of safety, lying several orders of magnitude above estimated human dietary exposures; However, this should be interpreted as evidence of toxicological tolerance under the specific conditions of this study, rather than as definitive proof of broad human safety. In this study, lab-made Clinoptilolite exhibited no evidence of systemic or genotoxic toxicity, reinforcing the view that zeolite minerals are inert and biologically stable when ingested. The study observations are consistent with published data on medicinal use of clinoptilolite, which is widely recognized as well tolerated following oral administration and has demonstrated potential for metal detoxification [13,14,15].

While the current study provides a comprehensive subacute safety evaluation, certain limitations should be acknowledged. The investigation was restricted to a 90-day exposure duration and a single rodent species, a single exposure rout, and limited dose spacing, which may limit generalizability of the findings to other species, exposure routes, or dose regimens. Although this design is adequate for determining a NOAEL in compliance with OECD Guidelines for testing of chemicals and for identifying systemic and genetic effects, longer-term or multigenerational, or mechanistic studies could provide additional insights into chronic, reproductive, or subtle biochemical effects. Moreover, some characterization data relied on unpublished sources, which may restrict reproducibility and external validation.

Despite these limitations, the study provides evidence that under the specific experimental conditions tested, the lab-made Clinoptilolite was well-tolerated and did not induce observable adverse effects. These findings support toxicological tolerance under controlled conditions, while recognizing that further studies involving multiple species, exposure routes, and fully published characterization data would be required to more comprehensively assess safety and generalizability.

## 5. Conclusions

This study investigated the safety profile of oral administration of the lab-made Clinoptilolite in its suspension form. Comprehensive toxicological evaluation of lab-made Clinoptilolite demonstrated an absence of adverse systemic or genotoxic effects under the conditions of these studies. Repeated oral administration at doses up to 15 mg/kg/day to Sprague Dawley rats produced no treatment-related changes in clinical observations, pathology, or histopathology. Minor variations in a few clinical parameters were incidental and lacked dose-dependent significance. All findings were reversible or within normal biological variation. Lab-made Clinoptilolite exhibited no mutagenic or clastogenic potential in series of genotoxicity testing, including the Ames assay, in vitro chromosomal aberration test, and in vivo micronucleus assay, all conducted in compliance with OECD and FDA guidance. Collectively, these results demonstrate that lab-made Clinoptilolite is well tolerated and non-toxic with the dose tested. When considered alongside adherence to internationally recognized testing guidelines and supportive evidence from similar aluminosilicate minerals, the data supports the conclusion that lab-made Clinoptilolite poses no observed systemic, genetic, or irreversible toxicological effects under the conditions tested. These findings contribute to a comprehensive toxicological profile which supports the characterization of lab-made Clinoptilolite as a candidate for GRAS status for its intended use as a food ingredient.

## Figures and Tables

**Table 1:** Summary of clinical signs across dose groups in the repeated-dose toxicity study.

Endpoint (Day 1–91)	Control	Clinoptilolite (mg/kg)		
		5	10	15
Mortality	0/10 (males), 0/10 (females)	0/10 (males), 0/10 (females)	0/10 (males), 0/10 (females)	0/10 (males), 0/10 (females)
Any clinical sign	Normal 10/10 (males), 10/10 (females)	No change 10/10 (males), 10/10 (females)	No change 10/10 (males), 10/10 (females)	No change 10/10 (males), 10/10 (females)
Detailed clinical exam	0/10 (males), 0/10 (females)	0/10 (males), 0/10 (females)	0/10 (males), 0/10 (females)	0/10 (males), 0/10 (females)
Functional Observation Battery	No treatment-related changes	No treatment-related changes	No treatment-related changes	No treatment-related changes
Ophthalmology (selected animals) *	No ocular abnormalities observed	No ocular abnormalities observed	No ocular abnormalities observed	No ocular abnormalities observed

Values represent the incidence of any abnormality by sex for each group (n = 10 per sex per group). * Assessments included: the cornea/anterior chamber, lens clarity, and the vitreous body/retina/optic disk.

**Table 2:** Terminal fasting body weight of male and female rats in repeated-dose toxicity study, group means ± SD (Day 91).

Group	Clinoptilolite (mg/kg/day)	Male		Females	
		FBW (Mean ± SD)	N	FBW (Mean ± SD)	N
Control	0	598.29 ± 37.73	10	309.22 ± 21.23	10
Low-dose	5	645.03 ± 46.27 *	10	314.65 ± 26.19	10
Mid-dose	10	624.71 ± 51.58	10	312.79 ± 15.81	10
High-dose	15	613.31 ± 55.77	10	312.79 ± 9.98	10

Values represent the group means ± SD at day 91. * Significantly different from control (*p* = 0.041). FBW: fasting body weight, SD: standard deviation.

**Table 3:** Weekly feed consumption (g/day) of male rats in repeated-dose toxicity study.

Interval	Control	Clinoptilolite (mg/kg/day)		
		5	10	15
Week 1 (Days 0–7)	28.4 ± 2.9	29.6 ± 3.1	29.0 ± 3.0	28.8 ± 3.4
Week 4 (Days 29–56)	30.8 ± 2.6	31.5 ± 3.0	31.2 ± 2.8	31.0 ± 3.1
Terminal interval (Days 85–91)	31.9 ± 2.8	32.5 ± 3.2	32.0 ± 2.9	31.6 ± 3.5

Values represent the mean ± SD, n = 10 (male).

**Table 4:** Weekly feed consumption (g/day) of female rats in repeated-dose toxicity study.

Interval	Control	Clinoptilolite (mg/kg/day)		
		5	10	15
Week 1 (Days 0–7)	16.1 ± 1.4	16.4 ± 1.6	16.2 ± 1.5	16.0 ± 1.3
Week 4 (Days 29–56)	17.0 ± 1.5	17.3 ± 1.6	17.2 ± 1.6	17.1 ± 1.4
Terminal interval (Days 85–91)	17.4 ± 1.6	17.6 ± 1.7	17.5 ± 1.6	17.3 ± 1.5

Values represent the mean ± SD, n = 10 (female).

**Table 5:** Hematology parameters of male rats in repeated-dose toxicity study (Day 91).

Parameter	Unit	Control	Clinoptilolite (mg/kg/day)		
			5	10	15
WBC	10^3^/µL	7.53 ± 1.36	7.85 ± 1.26	7.95 ± 1.02	7.88 ± 1.45
RBC	10^6^/µL	9.16 ± 0.46	8.58 ± 0.64 *	8.90 ± 0.57	8.98 ± 0.61
HGB	g/dL	15.3 ± 0.7	14.6 ± 0.9	14.9 ± 0.8	15.0 ± 0.9
HCT	%	48.2 ± 2.2	46.6 ± 2.9	47.1 ± 2.5	47.3 ± 2.6
MCV	fL	52.6 ± 1.8	54.3 ± 2.0	52.9 ± 1.7	52.7 ± 1.6
MCH	pg	16.7 ± 0.5	17.0 ± 0.6	16.8 ± 0.5	16.7 ± 0.6
MCHC	g/dL	31.8 ± 0.9	31.3 ± 0.8	31.7 ± 0.9	31.6 ± 0.8
PLT	10^3^/µL	810 ± 64	792 ± 78	820 ± 71	805 ± 67
RETIC	%	1.4 ± 0.3	1.5 ± 0.4	1.5 ± 0.3	1.4 ± 0.4
Neutrophils	%	17.3 ± 4.5	17.0 ± 4.9	17.7 ± 5.0	17.5 ± 4.8
Lymphocyte	%	76.2 ± 5.0	76.1 ± 5.3	76.9 ± 4.7	76.6 ± 4.8
Monocytes	%	3.58 ± 0.7	3.75 ± 0.6	3.62 ± 0.8	3.56 ± 0.6
Eosinophils	%	1.43 ± 0.4	1.42 ± 0.3	1.43 ± 0.4	1.44 ± 0.3
Basophils	%	0.0 ± 0.0	0.0 ± 0.0	0.0 ± 0.0	0.0 ± 0.0

Values represent the mean ± SD, n = 10 (male). * Significantly different from control (*p* = 0.037). WBC: white blood cell (count), RBC: red blood cell (count), HGB: hemoglobin, HCT: hematocrit, PLT: platelet (count), MCHC: mean corpuscular hemoglobin concentration.

**Table 6:** Hematology parameters of female rats in repeated-dose toxicity study (Day 91).

Parameter	Unit	Control	Clinoptilolite (mg/kg/day)		
			5	10	15
WBC	10^3^/µL	7.41 ± 1.58	7.80 ± 1.36	7.69 ± 1.21	7.45 ± 1.49
RBC	10^6^/µL	8.21 ± 0.47	8.05 ± 0.53	8.18 ± 0.61	8.07 ± 0.55
HGB	g/dL	13.2 ± 0.7	13.0 ± 0.8	13.3 ± 0.9	13.1 ± 0.8
HCT	%	40.1 ± 2.0	39.8 ± 2.1	40.3 ± 2.2	40.0 ± 2.0
MCV	fL	49.0 ± 1.6	49.3 ± 1.8	49.2 ± 1.7	49.1 ± 1.5
MCH	pg	16.1 ± 0.5	16.2 ± 0.5	16.3 ± 0.6	16.1 ± 0.6
MCHC	g/dL	32.8 ± 1.0	32.8 ± 1.1	33.0 ± 1.0	32.7 ± 1.0
PLT	10^3^/µL	680 ± 54	695 ± 61	672 ± 59	689 ± 56
RETIC	%	1.2 ± 0.3	1.3 ± 0.3	1.2 ± 0.3	1.2 ± 0.3
Neutrophils	%	17.92 ± 4.36	17.03 ± 4.72	16.13 ± 5.07	16.63 ± 4.91
Lymphocytes	%	76.72 ± 4.66	76.33 ± 5.37	78.05 ± 5.43	77.79 ± 5.83
Monocytes	%	3.92 ± 0.96	4.54 ± 0.61	3.88 ± 0.72	3.69 ± 1.08
Eosinophils	%	1.44 ± 0.38	2.1 ± 0.67 *	1.94 ± 0.66	1.89 ± 0.59
Basophils	%	0.0 ± 0.0	0.0 ± 0.0	0.0 ± 0.0	0.0 ± 0.0

Values represent the mean ± SD, n = 10 (female). * Significantly different from control (*p* < 0.05). WBC: white blood cell (count), RBC: red blood cell (count), HGB: hemoglobin, HCT: hematocrit, PLT: platelet (count), MCHC: mean corpuscular hemoglobin concentration.

**Table 7:** Clinical chemistry parameters of male and female rats in a repeated-dose toxicity study (Day 91).

Analyte (Unit)	Males				Females			
	Control	Clinoptilolite (5 mg/kg/day)	Clinoptilolite (10 mg/kg/day)	Clinoptilolite (15 mg/kg/day)	Control	Clinoptilolite (5 mg/kg/day)	Clinoptilolite (10 mg/kg/day)	Clinoptilolite (15 mg/kg/day)
Glucose (mg/dL)	103.11 ± 12.18	112.27 ± 9.13	116.65 ± 9.49 *	106.73 ± 9.15	112.4 ± 11.2	not reported	not reported	109.5 ± 12.8
Urea (BUN) (mg/dL)	29.23 ± 2.12	30.64 ± 4.47	30.64 ± 8.28	28.18 ± 3.51	27.9 ± 3.1	not reported	not reported	31.4 ± 4.0 *
Creatinine (mg/dL)	0.62 ± 0.04	0.62 ± 0.05	0.61 ± 0.05	0.63 ± 0.07	0.61 ± 0.05	—	—	—
ALT (U/L)	46.11 ± 4.81	45.95 ± 4.61	48.27 ± 7.01	47.52 ± 8.16	44.2 ± 4.5	not reported	not reported	45.8 ± 5.2
AST (U/L)	164.29 ± 25.82	152.95 ± 21.12	142.37 ± 19.83	144.07 ± 26.16	150.0 ± 20.0	not reported	not reported	142.4 ± 18.4
Total protein (TP) (g/L)	60.07 ± 1.94	61.00 ± 2.83	62.13 ± 1.77	63.56 ± 1.92 *	not reported	not reported	not reported	not reported
Albumin (ALB) (g/L)	29.04 ± 0.66	29.17 ± 0.81	29.93 ± 0.79	30.19 ± 1.27 *	not reported	not reported	not reported	not reported
HDL (mg/dL)	29.22 ± 7.50	26.03 ± 3.79	30.07 ± 6.08	27.09 ± 4.05	not reported	—	—	—
LDL (mg/dL)	22.88 ± 3.68	23.20 ± 3.57	23.85 ± 4.29	22.82 ± 3.77	not reported	—	—	—
Ca (mg/dL)	10.32 ± 0.16	10.45 ± 0.21	10.49 ± 0.44	10.64 ± 0.17 *	10.98 ± 0.39	10.89 ± 0.20	10.98 ± 0.52	11.03 ± 0.24
Na (mmol/L)	144.62 ± 1.63	142.08 ± 0.78 *	144.29 ± 2.38	143.32 ± 1.10	144.44 ±1.11	143.79 ± 1.11	144.13 ± 1.99	141.58 ± 1.99 *

Values represent the mean ± SD, n = 10 (male), n = 10 (female). * Significantly different from control (*p* < 0.05).

**Table 8:** Urinalysis parameters of male and female rats in repeated-dose toxicity study (Day 90).

Parameter	Unit	Control	Clinoptilolite (5 mg/kg/day)	Clinoptilolite (10 mg/kg/day)	Clinoptilolite (15 mg/kg/day)
Males					
Volume	mL/16 h	8.5 ± 2.4	8.1 ± 2.3	8.7 ± 2.1	8.4 ± 2.2
Specific gravity	—	1.042 ± 0.007	1.043 ± 0.006	1.041 ± 0.007	1.043 ± 0.006
pH	—	7.1 ± 0.3	7.2 ± 0.3	7.1 ± 0.4	7.2 ± 0.4
Protein	mg/dL	Trace–1+	Trace	Trace	Trace
Glucose	mg/dL	ND	ND	ND	ND
Ketones	mg/dL	ND	ND	ND	ND
Occult blood	—	ND	ND	ND	ND
Bilirubin	—	ND	ND	ND	ND
Urobilinogen	EU/dL	0.2 ± 0.1	0.2 ± 0.1	0.2 ± 0.1	0.2 ± 0.1
Microscopy	—	Occasional epithelial cells	Occasional	Occasional	Occasional
Females					
Volume	mL/16 h	6.9 ± 2.0	6.7 ± 1.8	7.2 ± 1.9	6.8 ± 2.1
Specific gravity	—	1.045 ± 0.006	1.044 ± 0.007	1.043 ± 0.007	1.045 ± 0.006
pH	—	7.0 ± 0.3	7.0 ± 0.4	7.1 ± 0.3	7.0 ± 0.3
Protein	mg/dL	Trace	Trace	Trace	Trace
Glucose	mg/dL	ND	ND	ND	ND
Ketones	mg/dL	ND	ND	ND	ND
Occult blood	—	ND	ND	ND	ND
Bilirubin	—	ND	ND	ND	ND
Urobilinogen	EU/dL	0.2 ± 0.1	0.2 ± 0.1	0.2 ± 0.1	0.2 ± 0.1
Microscopy	—	Occasional epithelial cells	Occasional	Occasional	Occasional

Values represent the mean ± SD, n = 10 (male), n = 10 (female).

**Table 9:** Absolute organ weights of male and female rats (Day 91).

Organ (g)/Endpoint	Male				Female			
	Control	Clinoptilolite (5 mg/kg/day)	Clinoptilolite (10 mg/kg/day)	Clinoptilolite (15 mg/kg/day)	Control	Clinoptilolite (5 mg/kg/day)	Clinoptilolite (10 mg/kg/day)	Clinoptilolite (15 mg/kg/day)
FBW	598.29 ± 37.73	645.03 ± 46.27 *	624.71 ± 51.58	613.31 ± 55.77	325.51 ± 26.83	309.22 ± 21.23	314.65 ± 26.19	312.79 ± 15.81
Liver	18.03 ± 1.43	22.70 ± 4.43 *	20.12 ± 2.07	20.58 ± 2.71	10.22 ± 1.71	9.58 ± 1.15	9.73 ± 1.21	9.98 ± 1.05
Kidneys	3.83 ± 0.50	4.09 ± 0.50	3.85 ± 0.53	4.04 ± 0.35	2.06 ± 0.20	2.03 ± 0.18	2.03 ± 0.20	2.12 ± 0.16
Spleen	0.93 ± 0.10	0.98 ± 0.09	0.98 ± 0.11	1.00 ± 0.16	0.58 ± 0.07	0.61 ± 0.08	0.58 ± 0.09	0.65 ± 0.10 *
Heart	1.98 ± 0.43	2.27 ± 0.33	2.10 ± 0.27	2.09 ± 0.31	1.18 ± 0.13	1.13 ± 0.12	1.14 ± 0.14	1.20 ± 0.20
Adrenals	—	—	—	—	0.09 ± 0.02	0.09 ± 0.01	0.09 ± 0.01	0.09 ± 0.02
Thymus	—	—	—	—	0.39 ± 0.07	0.35 ± 0.05	0.36 ± 0.07	0.36 ± 0.07
Brain	—	—	—	—	2.10 ± 0.16	1.98 ± 0.14	2.05 ± 0.16	1.97 ± 0.22
Testes	3.67 ± 0.43	3.89 ± 0.35	3.83 ± 0.27	3.96 ± 0.45	—	—	—	—
Ovaries	—	—	—	—	0.24 ± 0.05	0.22 ± 0.04	0.22 ± 0.05	0.23 ± 0.06
Uterus with cervix	—	—	—	—	0.79 ± 0.23	0.72 ± 0.20	0.72 ± 0.19	0.75 ± 0.16
Thyroid w/parathyroid	—	—	—	—	0.03 ± 0.00	0.03 ± 0.00	0.03 ± 0.00	0.03 ± 0.00
Pituitary gland	—	—	—	—	0.02 ± 0.00	0.02 ± 0.00	0.02 ± 0.00	0.02 ± 0.00

Values represent the mean ± SD, n = 10 (male), n = 10 (female). Organ weights were analyzed by ANCOVA with dose as a factor and terminal body weight as a covariate. Adjusted means and 95% confidence intervals are shown. * Significantly different from control (*p* < 0.05).

**Table 10:** Relative organ weights (% of terminal body weight) of male and female rats (Day 91).

Organ/Endpoint	Male				Female			
	Control	Clinoptilolite (5 mg/kg/day)	Clinoptilolite (10 mg/kg/day)	Clinoptilolite (15 mg/kg/day)	Control	Clinoptilolite (5 mg/kg/day)	Clinoptilolite (10 mg/kg/day)	Clinoptilolite (15 mg/kg/day)
Liver	3.01 ± 0.14	3.52 ± 0.24	3.22 ± 0.17	3.36 ± 0.19	3.14 ± 0.53	3.04 ± 0.38	3.10 ± 0.32	3.19 ± 0.68
Kidneys	0.64 ± 0.07	0.63 ± 0.05	0.62 ± 0.05	0.66 ± 0.07	0.67 ± 0.07	0.66 ± 0.04	0.65 ± 0.05	0.68 ± 0.07
Spleen	0.16 ± 0.02	0.15 ± 0.03	0.16 ± 0.03	0.16 ± 0.03	0.18 ± 0.02	0.20 ± 0.03	0.18 ± 0.02	0.21 ± 0.04 *
Heart	0.33 ± 0.02	0.35 ± 0.04	0.34 ± 0.03	0.34 ± 0.02	0.37 ± 0.03	0.37 ± 0.04	0.36 ± 0.03	0.38 ± 0.0
Brain	—	—	—	—	0.62 ± 0.04	0.66 ± 0.07	0.66 ± 0.06	0.63 ± 0.07
Thymus	—	—	—	—	0.11 ± 0.02	0.12 ± 0.02	0.12 ± 0.01	0.11 ± 0.02
Adrenals	—	—	—	—	0.03 ± 0.00	0.03 ± 0.00	0.03 ± 0.00	0.03 ± 0.01
Testes	0.613 ± 0.08	0.603 ± 0.07	0.613 ± 0.07	0.646 ± 0.07	—	—	—	—
Ovaries	—	—	—	—	0.06 ± 0.01	0.07 ± 0.01	0.07 ± 0.01	0.07 ± 0.02
Uterus	—	—	—	—	0.22 ± 0.03	0.23 ± 0.04	0.20 ± 0.02	0.24 ± 0.05
Thyroid	—	—	—	—	0.01 ± 0.00	0.01 ± 0.00	0.01 ± 0.00	0.01 ± 0.00
Pituitary gland	—	—	—	—	0.01 ± 0.00	0.01 ± 0.00	0.01 ± 0.00	0.01 ± 0.00

Values represent the mean ± SD and relative organ weights expressed as a percentage (%) of terminal body weight, n = 10 (male), n = 10 (female). * Significantly different from control (*p* < 0.05).

**Table 11:** Summary of microscopic findings and incidence by group in male and female rats.

Lesion	Tissue	Control	Clinoptilolite (mg/kg/day)			GLP Interpretation
			5	10	15	
Hepatocellular degeneration or necrosis	Liver	0/10	0/10	0/10	0/10	No treatment-related liver lesions.
Tubular degeneration	Kidney	≤1/10	≤1/10	≤2/10	≤2/10	Incidental/spontaneous; not dose-related.
Splenic extramedullary hematopoiesis	Spleen	≤1/10	≤1/10	≤2/10	≤2/10	Background finding.
Thyroid follicular changes	Thyroid	≤1/10	≤1/10	≤2/10	≤2/10	Not treatment-related.

Values represent the incidence of any abnormality by sex for each group (n = 10 per sex per dose group).

**Table 12:** Revertant colony counts (mean ± SD) from Trial 1 and Trial 2 under −S9 and +S9 conditions.

Strain	Condition	Trial 1 −S9	Trial 1 +S9	Trial 2 −S9	Trial 2 +S9	Interpretation
TA98	Vehicle	36.3 ± 1.5	37.0 ± 2.0	31.3 ± 3.1	33.3 ± 7.4	Baseline
	Test Item 5000 µg/plate	34.3 ± 1.5	37.0 ± 2.6	27.0 ± 1.0	29.7 ± 5.7	No increase vs. vehicle
	Positive control	494.7 ± 10.3 (2-NF)	504.7 ± 11.0 (2-AA)	494.7 ± 10.3 (2-NF)	500.0 ± 13.1 (2-AA)	Assay sensitivity confirmed
TA100	Vehicle	155.0 ± 5.3	157.3 ± 3.1	147.7 ± 5.5	147.3 ± 8.1	Baseline
	Test Item 5000 µg/plate	148.7 ± 3.2	153.3 ± 1.5	138.7 ± 3.1	137.0 ± 3.6	No increase
	Positive control	879.3 ± 54.0 (SA)	950.7 ± 56.1 (2-AA)	880.0 ± 13.1 (SA)	903.3 ± 12.1 (2-AA)	Marked increase
*E. coli* WP2 (pKM101)	Vehicle	160.7 ± 1.2	164.0 ± 3.6	162.0 ± 4.0	161.3 ± 3.1	Baseline
	Test Item 5000 µg/plate	154.3 ± 3.8	158.3 ± 3.5	150.0 ± 2.0	153.3 ± 6.4	No increase
	Positive control	866.7 ± 30.6 (4-NQO)	890.7 ± 31.4 (2-AA)	910.0 ± 14.4 (4-NQO)	898.0 ± 15.9 (2-AA)	Marked increase
TA1535	Vehicle	13.7 ± 1.5	14.3 ± 1.5	15.0 ± 3.0	14.7 ± 2.1	Baseline
	Test Item 5000 µg/plate	13.0 ± 1.0	13.7 ± 1.2	14.0 ± 4.0	13.0 ± 2.7	No increase
	Positive control	495.3 ± 15.0 (SA)	494.7 ± 11.7 (2-AA)	482.0 ± 4.0 (SA)	496.3 ± 7.5 (2-AA)	Marked increase
TA1537	Vehicle	11.7 ± 0.6	12.3 ± 2.1	13.0 ± 2.7	12.0 ± 1.0	Baseline
	Test Item 5000 µg/plate	10.7 ± 1.2	12.0 ± 1.0	11.3 ± 2.3	11.3 ± 1.5	No increase
	Positive control	482.7 ± 3.1 (ICR-191)	461.3 ± 41.4 (2-AA)	465.3 ± 13.0 (ICR-191)	478.6 ± 14.5 (2-AA)	Marked increase

Values represent the mean ± SD. Study population/units: Bacterial plates; three replicate plates per treatment condition as per OECD; no animals.

**Table 13:** In vivo micronucleus test results (OECD 474).

Group	Dose (mg/kg)	Mean % MnPCE ± SD	Mean % PCE ± SD	N (main)
Vehicle control	0	2.7 ± 1.06	47.3 ± 2.5	10
Test item	2000	4.0 ± 1.49	46.2 ± 3.0	10
Positive control (CPA)	50	15.3 ± 1.06	39.4 ± 2.7	10

N = 10 (5 male and 5 female mice) per group.

**Table 14:** Pooled body weight and feed consumption of male and female rats during the recovery period.

Endpoint	Recovery Group (Post-Treatment)		Interpretation
	Control	Clinoptilolite (15 mg/kg/day)	
Terminal BW (g)	594.1 ± 36.2	603.2 ± 39.5	No persistent difference observed; values comparable.
Feed consumption (g/day)	30.5 ± 2.4	30.9 ± 2.6	Returned to control levels.

Values represent the mean ± SD, n = 10 (5 male and 5 female rats) per group. BW = body weight.

**Table 15:** Pooled hematology and clinical chemistry parameters of male and female rats during the recovery period.

Parameter	Recovery Group (Post-Treatment)		GLP Interpretation
	Control	Clinoptilolite (15 mg/kg/day)	
ALT (U/L)	42.1 ± 4.2	43.3 ± 5.1	Values comparable; earlier terminal increases resolved.
BUN (mg/dL)	28.5 ± 3.1	29.1 ± 3.5	No persistent change.
Creatinine (mg/dL)	0.61 ± 0.05	0.62 ± 0.06	—

Values represent the mean ± SD, 10 (5 male and 5 female rats) per group. ALT: Alanine Aminotransferase, BUN: Blood Urea Nitrogen.

**Table 16:** Pooled organ weights and histopathology findings of male and female rats during the recovery period.

Organ/Finding	Recovery Group (Post-Treatment)		GLP Interpretation
	Control	Clinoptilolite (15 mg/kg/day)	
Liver absolute (g)	18.0 ± 1.5	18.5 ± 1.7	No persistent differences after recovery.
Liver histopathology incidence	0/10	0/10	No persistent lesions attributable to treatment.
Kidney absolute (g)	3.9 ± 0.4	4.0 ± 0.5	No persistent differences after recovery.

Values represent the mean ± SD and the incidence of any abnormality for each group, 10 (5 male and 5 female rats) per group.

## Data Availability

The datasets generated and analyzed during the current study are available from the corresponding author upon reasonable request.

## Supplementary Materials

The following supporting information can be downloaded at: https://www.mdpi.com/article/10.3390/toxics14020122/s1. Figure S1: Quantitative phase identification for commercially available mined clinoptilolite A; Figure S2: Quantitative phase identification for commercially available mined clinoptilolite B; Figure S3:Quantitative phase identification for lab-made clinoptilolite powder A; Figure S4: Quantitative phase identification for lab-made clinoptilolite powder B; Figure S5: Quantitative phase identification for lab-made clinoptilolite nano-suspension A; Figure S6: Quantitative phase identification for lab-made clinoptilolite nano-suspension B.

## References

[1] Ambrozova P., Kynicky J., Urubek T., Nguyen V.D. (2017). Synthesis and Modification of Clinoptilolite. Molecules.

[2] Doula M.K., Elaiopoulos K., Kavvadias V.A., Mavraganis V. (2012). Use of Clinoptilolite to Improve and Protect Soil Quality from the Disposal of Olive Oil Mills Wastes. J. Hazard. Mater..

[3] Dosa M., Grifasi N., Galletti C., Fino D., Piumetti M. (2022). Natural Zeolite Clinoptilolite Application in Wastewater Treatment: Methylene Blue, Zinc and Cadmium Abatement Tests and Kinetic Studies. Materials.

[4] Eroglu N., Emekci M., Athanassiou C.G. (2017). Applications of Natural Zeolites on Agriculture and Food Production. J. Sci. Food Agric..

[5] Mastinu A., Kumar A., Maccarinelli G., Bonini S.A., Premoli M., Aria F., Gianoncelli A., Memo M. (2019). Zeolite Clinoptilolite: Therapeutic Virtues of an Ancient Mineral. Molecules.

[6] Markoska R., Stojković R., Filipović M., Jurin M., Špada V., Kavre Piltaver I., Pavelić K., Marković D., Kraljević Pavelić S. (2023). Study of Zeolite Clinoptilolite d-Glucose Adsorption Properties In Vitro and In Vivo. Chem. Biol. Interact..

[7] Kraljević Pavelić S., Simović Medica J., Gumbarević D., Filošević A., Pržulj N., Pavelić K. (2018). Critical Review on Zeolite Clinoptilolite Safety and Medical Applications In Vivo. Front. Pharmacol..

[8] Basha M.P., Begum S., Mir B.A. (2013). Neuroprotective Actions of Clinoptilolite and Ethylenediaminetetraacetic Acid Against Lead-Induced Toxicity in Mice Mus musculus. Toxicol. Int..

[9] Ates A., Hardacre C. (2012). The Effect of Various Treatment Conditions on Natural Zeolites: Ion Exchange, Acidic, Thermal and Steam Treatments. J. Colloid Interface Sci..

[10] Senila M., Cadar O. (2024). Modification of Natural Zeolites and Their Applications for Heavy Metal Removal from Polluted Environments: Challenges, Recent Advances, and Perspectives. Heliyon.

[11] Organisation for Economic Co-operation and Development (OECD) (2023). OECD Guidelines for the Testing of Chemicals: Tests 471, 473, 474; Bacterial Reverse Mutation Test, In Vitro Mammalian Chromosome Aberration Test, Mammalian Erythrocyte Micronucleus Test.

[12] US Food and Drug Administration (2023). Toxicological Principles for the Safety Assessment of Food Ingredients.

[13] Dolanc I., Ferhatović Hamzić L., Orct T., Micek V., Šunić I., Jonjić A., Jurasović J., Missoni S., Čoklo M., Pavelić S.K. (2023). The Impact of Long-Term Clinoptilolite Administration on the Concentration Profile of Metals in Rodent Organisms. Biology.

[14] Kraljević Pavelić S., Micek V., Filošević A., Gumbarević D., Žurga P., Bulog A., Orct T., Yamamoto Y., Preočanin T., Plavec J. (2017). Novel, Oxygenated Clinoptilolite Material Efficiently Removes Aluminium from Aluminium Chloride-Intoxicated Rats In Vivo. Microporous Mesoporous Mater..

[15] Souza I.M.S., García-Villén F., Viseras C., Perger S.B.C. (2023). Zeolites as Ingredients of Medicinal Products. Pharmaceutics.

