# Supplementary material for: Safety Evaluation of Lab-Made Clinoptilolite: 90-Day Repeated Dose Toxicity Study in Sprague Dawley Rats and a Battery of In Vitro and In Vivo Genotoxicity Tests
Source: Toxics. 2026 Jan 28;14(2):122. doi: 10.3390/toxics14020122 (PMC12944921; doi:10.3390/toxics14020122)
Supplement: Supplementary file 1 [file toxics-14-00122-s001.zip › toxics-4062004-supplementary.pdf]

Supplementary Materials

Experimental Details

All characterization was performed using Rigaku SmartLab Diffractometer XE. Samples were lightly ground in a mortar and pestle before measurement to improve phase identification. In Bragg–Brentano geometry, symmetric Theta-2Theta scans were collected for analysis with the following parameters: scan range 5 – 60°, step size 0.02°, scan speed 3°/min. Data was matched against the ICDD PDF-4+ database. Estimated error in weight percentages is 5 – 10 %.

XRD Characterization of Commercially Available Mined Clinoptilolite

Figure S1. Quantitative phase identification for commercially available mined clinoptilolite A.

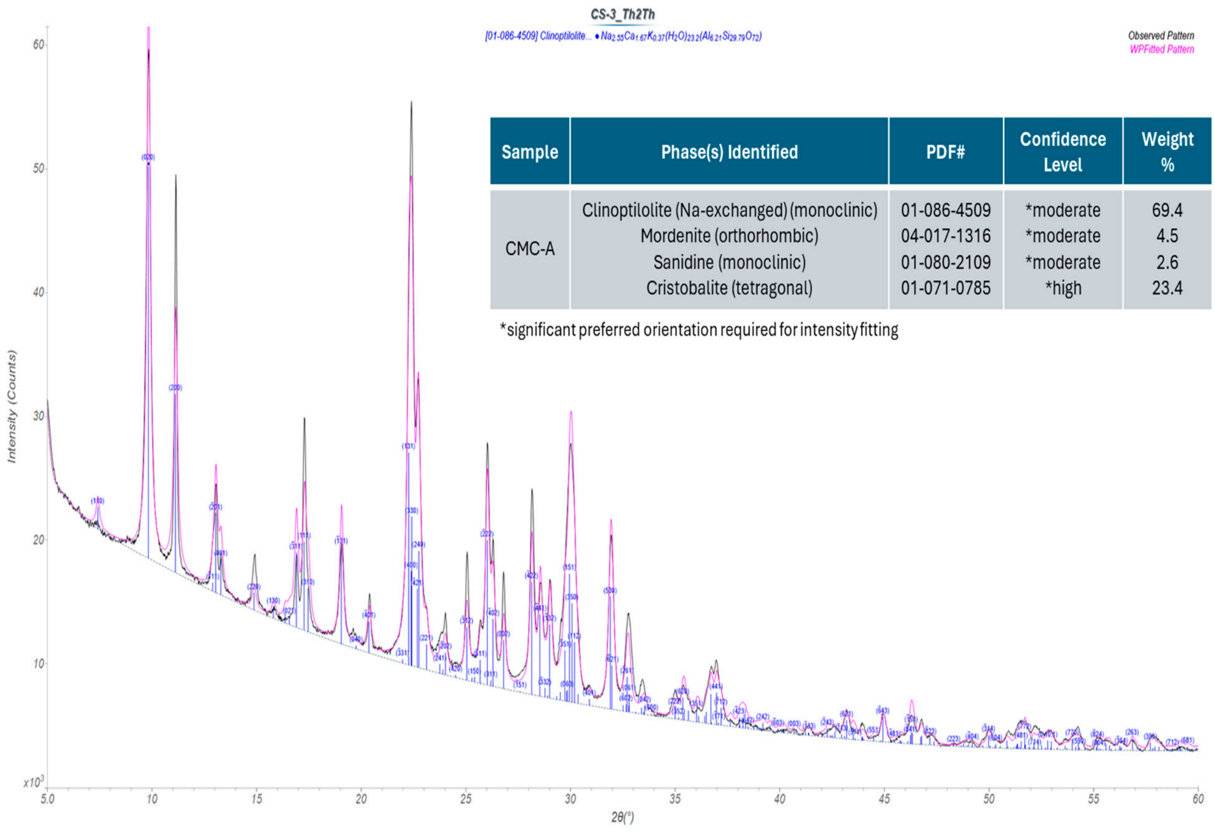

Sample	Phase(s) Identified	PDF#	Confidence Level	Weight %
CMC-A	Clinoptilolite (Na-exchanged) (monoclinic)<br>Mordenite (orthorhombic)<br>Sanidine (monoclinic)<br>Cristobalite (tetragonal)	01-086-4509<br>04-017-1316<br>01-080-2109<br>01-071-0785	*moderate<br>*moderate<br>*moderate<br>*high	69.4<br>4.5<br>2.6<br>23.4

*significant preferred orientation required for intensity fitting

Figure S2. Quantitative phase identification for commercially available mined clinoptilolite B.

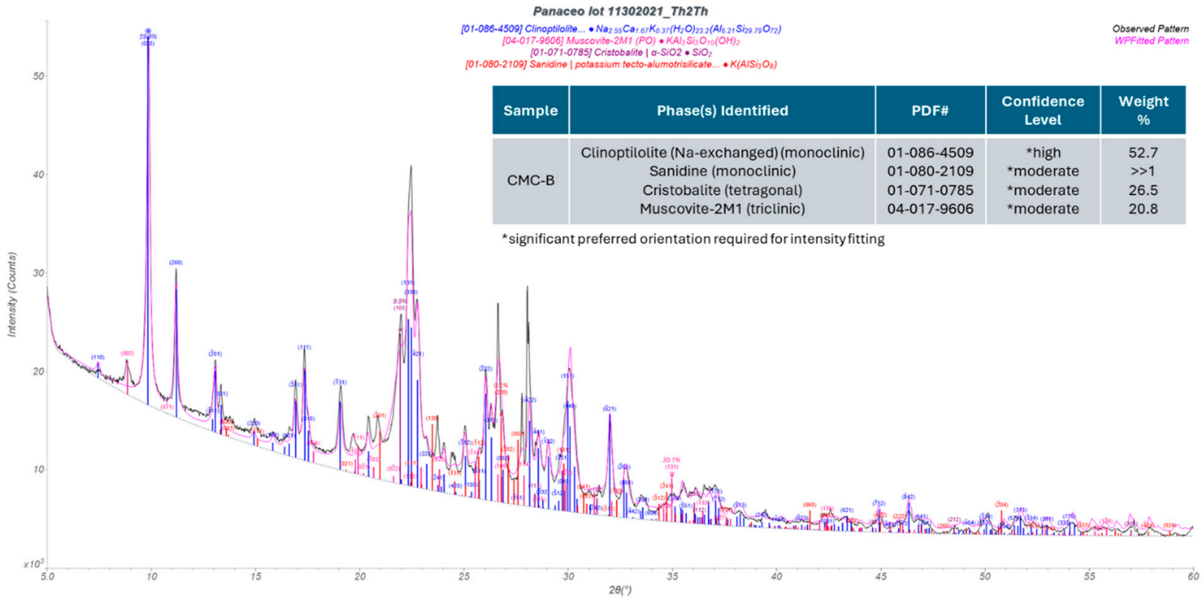

Sample	Phase(s) Identified	PDF#	Confidence Level	Weight %
CMC-B	Clinoptilolite (Na-exchanged) (monoclinic)<br>Sanidine (monoclinic)<br>Cristobalite (tetragonal)<br>Muscovite-2M1 (triclinic)	01-086-4509<br>01-080-2109<br>01-071-0785<br>04-017-9606	*high<br>*moderate<br>*moderate<br>*moderate	52.7<br>>>1<br>26.5<br>20.8

*significant preferred orientation required for intensity fitting

XRD Characterization of Lab-Made Clinoptilolite Powder

Figure S3. Quantitative phase identification for lab-made clinoptilolite powder A.

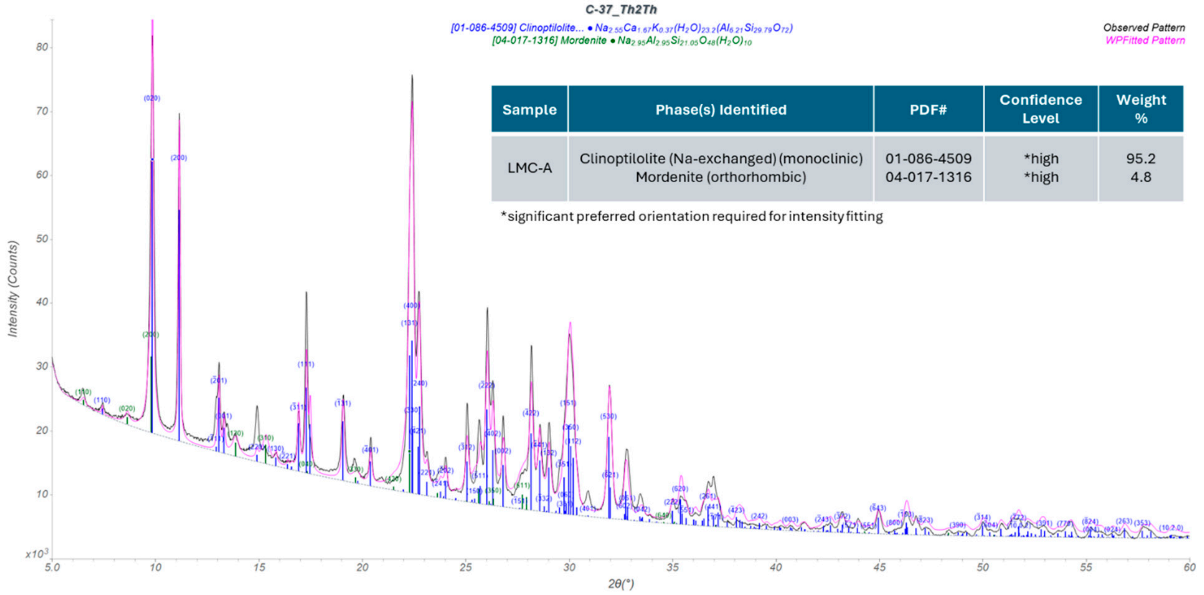

Sample	Phase(s) Identified	PDF#	Confidence Level	Weight %
LMC-A	Clinoptilolite (Na-exchanged) (monoclinic)<br>Mordenite (orthorhombic)	01-086-4509<br>04-017-1316	*high<br>*high	95.2<br>4.8

*significant preferred orientation required for intensity fitting

Figure S4. Quantitative phase identification for lab-made clinoptilolite powder B.

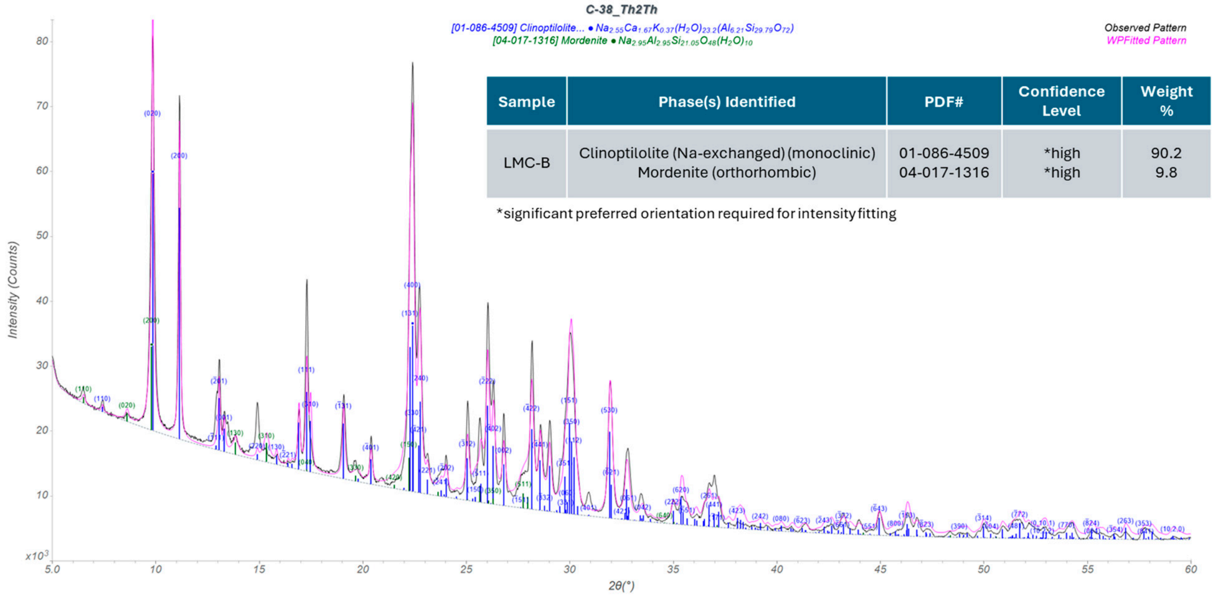

Sample	Phase(s) Identified	PDF#	Confidence Level	Weight %
LMC-B	Clinoptilolite (Na-exchanged) (monoclinic)<br>Mordenite (orthorhombic)	01-086-4509<br>04-017-1316	*high<br>*high	90.2<br>9.8

*significant preferred orientation required for intensity fitting

XRD Characterization of Lab-Made Clinoptilolite Nano-Suspension

A powder sample for XRD was obtained by drying the nano-suspension at 230 deg F overnight.

Figure S5. Quantitative phase identification for lab-made clinoptilolite nano-suspension A.

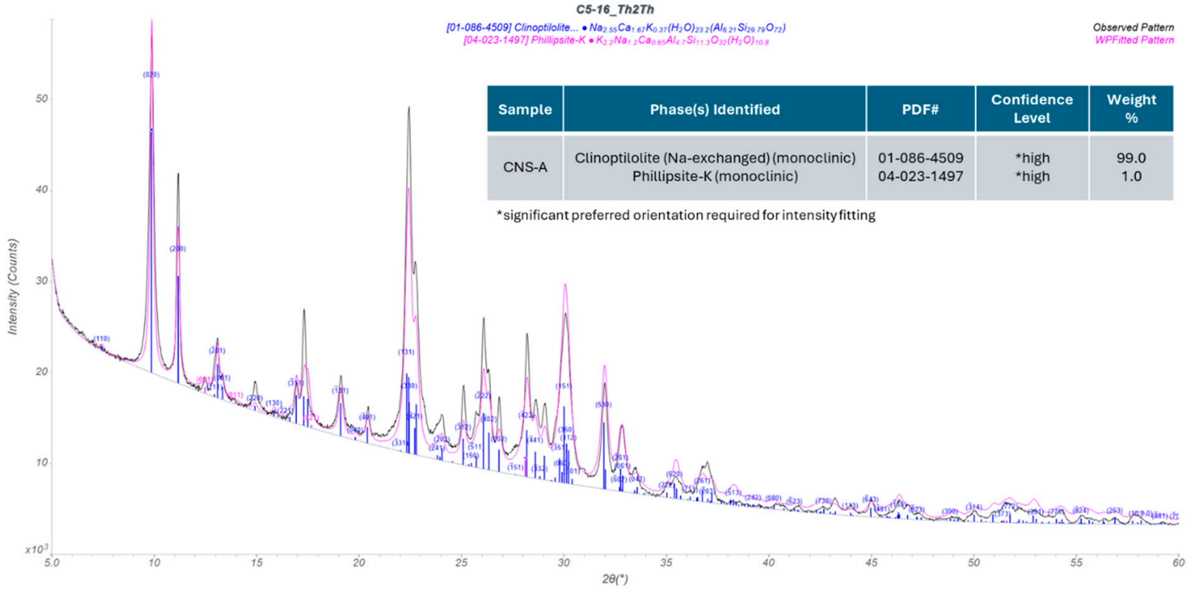

Sample	Phase(s) Identified	PDF#	Confidence Level	Weight %
CNS-A	Clinoptilolite (Na-exchanged) (monoclinic)<br>Phillipsite-K (monoclinic)	01-086-4509<br>04-023-1497	*high<br>*high	99.0<br>1.0

*significant preferred orientation required for intensity fitting

Figure S6. Quantitative phase identification for lab-made clinoptilolite nano-suspension B.

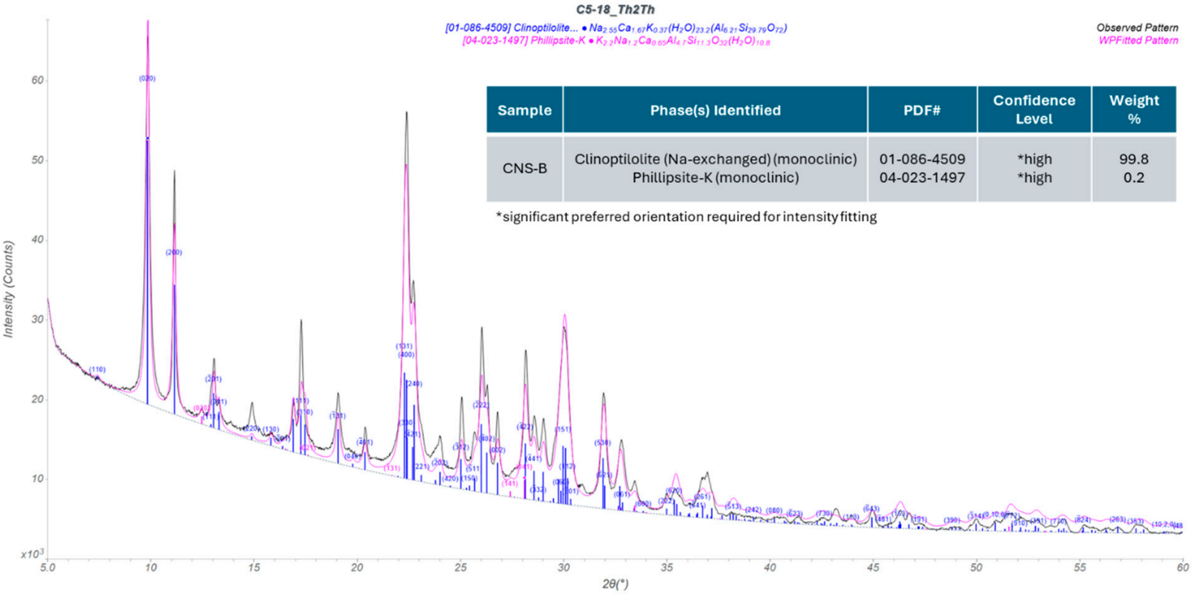

Sample	Phase(s) Identified	PDF#	Confidence Level	Weight %
CNS-B	Clinoptilolite (Na-exchanged) (monoclinic)<br>Phillipsite-K (monoclinic)	01-086-4509<br>04-023-1497	*high<br>*high	99.8<br>0.2

*significant preferred orientation required for intensity fitting
